# The linkers in fluorene-labeled 2′-deoxyuridines affect fluorescence discriminating phenomena upon duplex formation†

**DOI:** 10.1039/d0ra01651a

**Published:** 2020-05-19

**Authors:** So Young Lee, Seung Woo Hong, Hyeonuk Yeo, Gil Tae Hwang

**Affiliations:** Department of Chemistry and Green-Nano Materials Research Center, Kyungpook National University Daegu 41566 Republic of Korea giltae@knu.ac.kr; Department of Chemistry Education, Kyungpook National University Daegu 41566 Republic of Korea yeo@knu.ac.kr

## Abstract

Three fluorene-labeled 2′-deoxyuridines that differ in terms of their linkers—U^F^ (without linker), U^FL^ (with ethynyl linker), and U^DF^ (with diethynyl linker)—have been introduced at the central positions of oligodeoxynucleotides to examine the effects that their linkers have on the fluorescence emission properties upon duplex formation with fully matched and single-base-mismatched targets. Here, we describe the influence of the linkers on the emission behavior, the intramolecular electron transfer between the fluorene moiety and the uracil base after photoexcitation, and the structural stability upon duplex formation. The probe containing the U^FL^ residue (with an ethynyl linker) and cytosine residues as flanking bases exhibited the greatest fluorescence turn-on selective behavior toward the perfectly matched target.

## Abstract 2

Three fluorene-labeled 2′-deoxyuridines that differ in terms of their linkers have been introduced at the central positions of oligodeoxynucleotides to examine the effects that their linkers have on the emission properties upon duplex formations.

## Graphical abstract

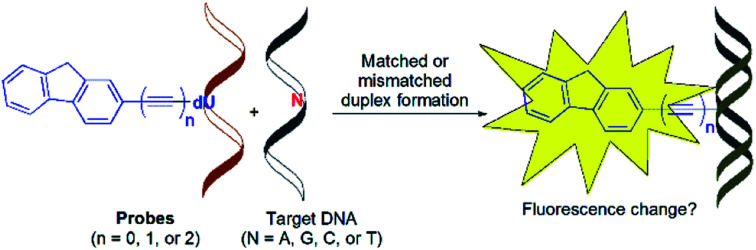

## Introduction

**Fig. 1:**
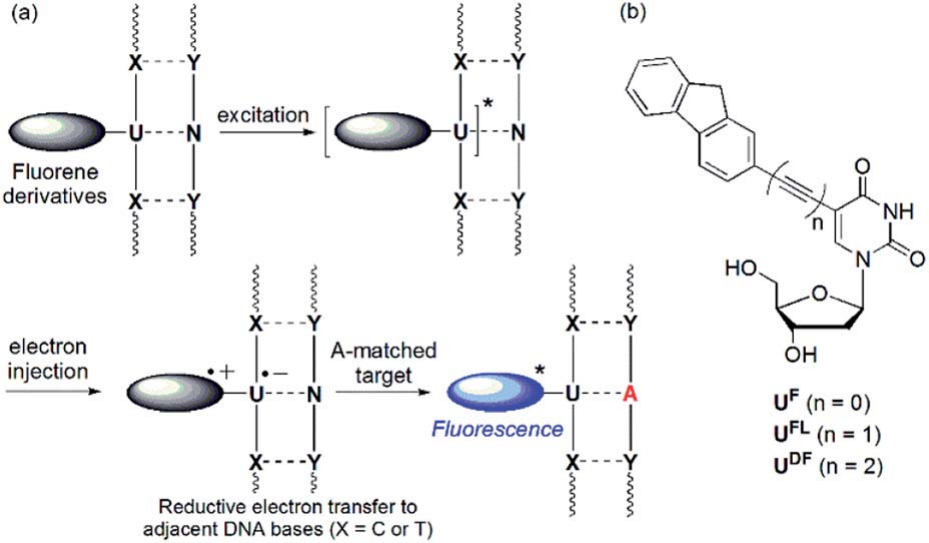
(a) Mechanism of operation of quencher-free MB systems. (b) Structures of fluorene-labeled 2′-deoxyuridines featuring various linkers.

Detection of single nucleotide polymorphisms (SNPs) is an important aspect of the identification and diagnosis of disease-causing genes.^1^ Molecular beacons (MBs) featuring fluorophore and quencher units at the ends of their stems are used widely for the detection and analysis of SNPs.^2^ In the absence of a specific target, the emission of an MB is quenched as a result of the proximity of the fluorophore and quencher units; in contrast, duplex formation with the target nucleic acid results in a large increase in the fluorescence intensity. Interestingly, sequence-specific detection of nucleic acids is also possible when using fluorescently labeled oligonucleotide probes that feature no quencher unit.^3^ Recently, we developed a quencher-free MB system in which a 2′-deoxyuridine residue was linked to fluorene derivatives as labels through an ethynyl linker.^4,5^ That system could distinguish, through changes in fluorescence intensity, perfectly matched from single-base-mismatched sequences. Based on differences in the reducibility of nucleobases, intramolecular charge transfer (ICT) from fluorene derivatives to flanking pyrimidine residues (C or T) upon photoexcitation results in efficient quenching of single-stranded oligodeoxynucleotides (ssODNs) and single-base mismatched double-stranded ODNs (Fig. 1a).^6^ In addition, ODNs featuring C-flanking bases (C-FBs) can display additionally decreased fluorescence when hybridized with all-mismatched targets, when compared with that of ssODNs. This dramatic quenching arises because pairs of guanine bases (*i.e.*, the complementary bases of C-FBs) act as internal quenchers when approached by the fluorene derivative.^7^ When the ODN encounters a perfectly matched target, however, this quenching is inhibited and the emission of the fluorene derivative is restored. Because an acetylene unit linking the fluorene derivatives to the 2′-deoxyuridine residue is efficient at mediating these electron transfer processes, fluorophore-labeled deoxyuridines featuring ethynyl linkers have been studied widely as fluorescent probes.^8^

The ICT process is dependent on two features: (i) the energy difference between the donor's highest occupied molecular orbital (HOMO) and the acceptor's lowest unoccupied molecular orbital (LUMO) and (ii) the nature of the spacer mediating electron transfer between the two groups. In this study, we investigated how SNP probes would operate in the absence of a linker, and when a diethynyl unit was used as an alternative to an ethynyl moiety, linking the fluorene and 2′-deoxyuridine moieties (Fig. 1b). In previous studies, 2′-deoxyuridine residues labeled with chromophores through by a single C–C bond (*i.e.*, linker-free systems) have been developed as fluorescence turn-on probes for the detection of an abasic site,^9^ triple-helix formation,^10^ a matched adenosine target^11^ and isomorphic thymine analogues.^12^ The only previously reported example of a DNA probe featuring a 2′-deoxyuridine residue labeled with a diethynyl linker was described by the Brown group,^13^ but their anthracene-labeled 2′-deoxyuridine residue featuring a diacetylenic unit could not discriminate effectively between matched and one-base-mismatched targets through changes in fluorescence intensity.

## Results and discussion

**Scheme 1:**
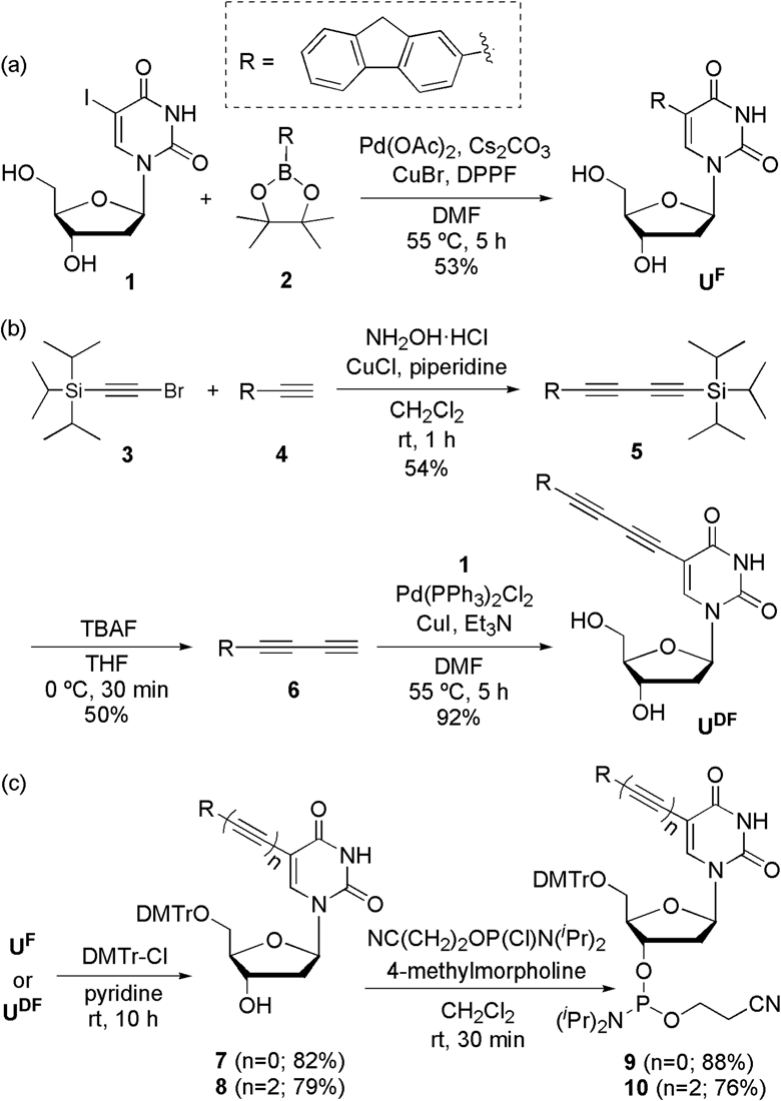
Synthesis of (a) U^F^, (b) U^DF^ and (c) their phosphoramidites 9 and 10, respectively.

U^F^ and U^DF^ were synthesized from 2′-deoxy-5-iodouridine (1) through a Suzuki reaction (Scheme 1a) with fluorene-2-boronic acid pinacol ester (2) and a Sonogashira reaction (Scheme 1b) with 2-(buta-1,3-diynyl)fluorene (6), respectively. U^FL^ was synthesized using previously reported procedures.^4*f*^ To obtain 2-(buta-1,3-diynyl)fluorene (6), we performed Cadiot–Chodkiewicz coupling^14^ of (bromoethynyl)triisopropylsilane (3)^15^ and 2-ethynylfluorene (4),^16^ followed by deprotection of the triisopropylsilyl group using tetrabutylammonium fluoride. After protecting the OH groups of U^F^ and U^DF^ with DMTr units, we converted the nucleosides to the phosphoramidites 9 and 10, respectively (Scheme 1c).

Initially, we measured the absorption and emission spectra of the free nucleosides in MeOH (Table 1 and Fig. S1†). U^F^ exhibited the highest absorbance among our three tested nucleosides; U^FL^ displayed red-shifted absorption and emission signals relative to those of the more highly conjugated U^DF^. To determine the causes of these phenomena, we employed time-dependent density functional theory (TDDFT) to calculate the optimized structures of the ground and excited states of these nucleosides *in vacuo* (Fig. S2 and S3†). The dihedral angles between the fluorene and uracil moieties in the optimized structures of the ground states were 23.29° for U^F^, 0.77° for U^FL^ and 10.21° for U^DF^; in their excited states they were 4.94, 3.95 and 26.87°, respectively. For both U^F^ and U^DF^, the absorption maximum was blue-shifted relative to that of U^FL^ because the dihedral angles between their fluorene and uracil moieties were twisted in the ground state (*i.e.*, less effective conjugation). In addition, the emission maximum of U^DF^ was similar to that of U^F^ (which featured a planar dihedral angle in the excited state) and much more blue-shifted than that of U^FL^, because U^DF^ remained relatively twisted in its excited state. This twist was presumably responsible for the low fluorescence yield of the U^DF^.

**Table 1:** Photophysical data for U^F^, U^FL^ and U^DF^ in MeOH at 25 °C

Nucleoside	*λ*_max_/nm^a^	*ε*/M^−1^ cm^−1^	*λ*_em_/nm^b^	*Φ*_F_ c
U^F^	310	89 000	419	0.16
U^FL^^d^	369	25 000	453	0.26
U^DF^	363	26 000	418	0.022

a Only the largest absorption maxima are listed.

b Wavelength of emission maximum when excited at the absorption maximum.

c Quantum efficiencies determined using a cyclohexane solution of pyrene (*λ*_ex_ = 313 nm) for U^F^, a solution of quinine sulfate in 0.1 N H_2_SO_4_ (*λ*_ex_ = 350 nm) for U^FL^ and a solution of fluorescein in 0.1 N NaOH (*λ*_ex_ = 366 nm) for U^DF^ as standards.^18^ Data are presented as mean values from three independent experiments.

d Taken from ref. 17.

We used a DNA synthesizer and the phosphoramidites 9 and 10 to incorporate the U^F^ and U^DF^ residues, respectively, at the central positions of ODNs (Table 2). We characterized these ODNs using matrix-assisted laser desorption ionization time-of-flight (MALDI-TOF) mass spectrometry (Table S1†). To determine the suitability of these ODNs as SNP probes, we positioned pyrimidine bases (C or T) as FBs for the U^F^ and U^DF^ residues and compared their effects with those previously reported for U^FL^ residues.

**Table 2:** ODNs tested in this study

ODN^a^	Sequence
ODN1(X)	5′-d(TGG ACT TXT TCA ATG)-3′
ODN1′(N)	3′-d(ACC TGA ANA AGT TAC)-5′
ODN2(X)	5′-d(TGG ACT CXC TCA ATG)-3′
ODN2′(N)	3′-d(ACC TGA GNG AGT TAC)-5′

a X: U^F^, U^FL^ or U^DF^; N: A, T, G or C.

**Fig. 2:**
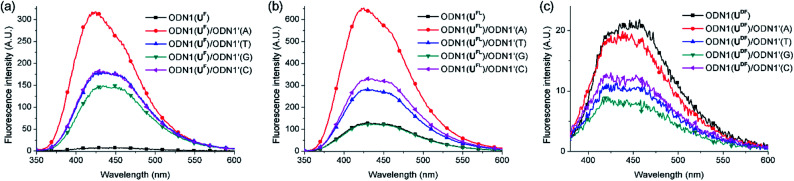
Fluorescence spectra of probes featuring T-FBs, recorded in 10 mM Tris–HCl buffer (pH 7.2; 100 mM NaCl, 20 mM MgCl_2_): ssODN1s and their duplexes (each concentration: 1.5 μM) formed between (a) ODN1(U^F^)/ODN1′(N), (b) ODN1(U^FL^)/ODN1′(N) and (c) ODN1(U^DF^)/ODN1′(N); N = A, T, G or C. Excitation wavelength: absorption maximum.

First, we investigated the discrimination against single-nucleotide variants exhibited by the fluorescent ODN1 featuring T-FBs (Fig. 2). When the ODN1s containing U^F^ and U^FL^ residues formed their matched duplexes, their emissions were enhanced 37 and 4.6 times, respectively, relative to those of the ssODN1s (Table 3). Notably, the fluorescence of ODN1(U^F^) itself was almost completely quenched through effective ICT; it recovered completely upon formation of its matched duplex. ODN1(U^FL^), featuring an acetylenic linker, did not experience ICT as effective as that of U^F^. Furthermore, when forming mismatched duplexes, the ODN1s containing U^F^ and U^FL^ residues provided fluorescence increases greater than those of the ssODN1s, in most cases, making it difficult to use them as A-selective SNP probes. All of the matched and mismatched duplexes of ODN1(U^DF^) displayed emissions slightly lower than those of ssODN1(U^DF^). These results indicate that ODN1(U^DF^) featuring T-FBs and diacetylenic linker did not produce effective ICT or its formation of duplex with A-matched target did not inhibit fluorescence quenching caused by the ICT process.

**Table 3:** Total discrimination factors of ODN1 bearing T-FBs and ODN2 bearing C-FBs^a^

Duplex^b^	U^F^	U^FL^^c^	U^DF^
ODN1(X)/ODN1′(A)	37	4.6	0.91
ODN1(X)/ODN1′(T)	23	2.1	0.54
ODN1(X)/ODN1′(G)	19	0.96	0.43
ODN1(X)/ODN1′(C)	23	2.5	0.63
ODN2(X)/ODN2′(A)	5.7	4.0	2.6
ODN2(X)/ODN2′(T)	1.6	0.39	0.72
ODN2(X)/ODN2′(G)	1.1	0.35	0.67
ODN2(X)/ODN2′(C)	2.9	0.27	0.91

a Area ratio of fluorescence intensity relative to those of corresponding ssODNs.

b X: U^F^, U^FL^ or U^DF^.

c Taken from ref. 4*a*.

**Fig. 3:**
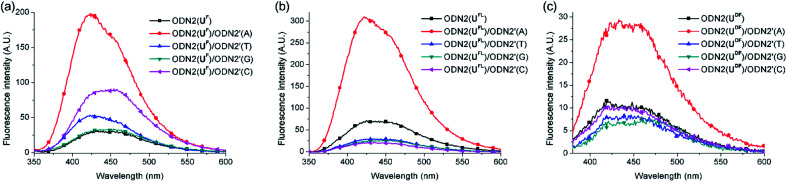
Fluorescence spectra of probes featuring C-FBs, recorded in 10 mM Tris–HCl buffer (pH 7.2; 100 mM NaCl, 20 mM MgCl_2_): ssODN2s and their duplexes (each concentration: 1.5 μM) formed between (a) ODN2(U^F^)/ODN2′(N), (b) ODN2(U^FL^)/ODN2′(N) and (c) ODN2(U^DF^)/ODN2′(N); N = A, T, G or C. Excitation wavelength: absorption maximum.

Next, we investigated the fluorescence behavior of the ODN2 featuring C-FBs (Fig. 3). The fluorescence of the matched duplex of ODN2(U^FL^) was four-fold greater than that of ssODN2(U^FL^); the mismatched duplexes exhibited decreases in emission that were more than twice that of ssODN, resulting from the quinine-quenching effect (G-effect). Similar to the situation for ODN1(U^DF^) having T-FBs, ODN2(U^DF^) featuring C-FBs displayed decreased fluorescence relative to that of ssODN when forming duplexes with mismatched targets. However, the formation of duplex with A-matched target showed only a 2.6-fold increase in fluorescence relative to that of ssODN2(U^DF^). In other words, the diacetylenic linker did not facilitate effective ICT. Interestingly, when ODN2(U^F^) formed its matched duplex, there was a 5.7-fold increase in fluorescence relative to that of ssODN2(U^F^)—slightly higher than the 4.0-fold increase of ssODN2(U^FL^). Thus, it appeared that the linker-free U^F^ residue was slightly more suitable for effective electronic coupling between the fluorene and uracil moieties for ICT than was the U^FL^ residue featuring an acetylenic linker. Nevertheless, ODN2(U^F^) exhibited slightly increased emissions upon hybridization with all of its mismatched targets, resulting in 1.6 (T)-, 1.1 (G)- and 2.9 (C)-fold enhancements in its emission intensities relative to that of ssODN2(U^F^). That is, the G-effect observed for ODN2(U^FL^) did not operate for ODN2(U^F^). We suspect that the fluorene unit was located too close to the duplexes of ODN2(U^F^), such that partial distortion of the duplex structure (or of the dihedral angle between the fluorene and uracil moieties) occurred when forming mismatched duplexes; thus, it was less likely that the G-effect or ICT through photoexcitation could be generated. We measured melting temperatures to compare the thermal stabilities of the duplexes formed using the ODN2s (Table 4). The A-selective thermal stability observed for the duplexes of ODN2(U^FL^) was not evident for ODN2(U^F^) and ODN2(U^DF^). That is, the values of *T*_m_ for the mismatched duplexes of ODN2(U^F^) and ODN2(U^DF^) were higher than those of ODN2(U^FL^). This phenomenon was presumably caused by the structural modification of the U^F^ and U^DF^ residues, and additionally weaker G-effects, making them ineffective DNA probes.

**Table 4:** Melting temperatures (*T*_m_) of duplexes^a^

Duplex	X = T	X = U^F^	X = U^FL^	X = U^DF^
ODN2(X)/ODN2′(A)	57.3	48.3	48.7	49.5
ODN2(X)/ODN2′(T)	49.4	46.9	42.8	46.5
ODN2(X)/ODN2′(G)	51.8	45.6	43.6	47.8
ODN2(X)/ODN2′(C)	47.1	47.8	45.8	47.9

a All values of *T*_m_ (1.5 μM) were measured in 10 mM Tris–HCl buffer (100 mM NaCl, 20 mM MgCl_2_; pH 7.2) and are provided as averages from three independent measurements.

In summary, effective electron transfer between the two moieties occurred when the fluorene and uracil units were connected directly without a linker. This tendency was more pronounced when T-FBs were involved, resulting in a 37-fold increase in fluorescence when forming duplexes with A-matched targets. The application of a U^F^ residue, however, was not suitable for discriminating mismatched duplexes. In addition, ODNs featuring a U^DF^ residue, bearing a diacetylenic linker, were not useful as SNP probes because of low quantum yields and no electron transfer between the fluorene and uracil moieties, due to their twisted dihedral angle in the excited state. The U^FL^ residue, bearing an ethynyl linker, did, however, feature an appropriate distance between the fluorene and uracil moieties to ensure effective electron transfer and structural stability of its duplexes. Nevertheless, the electron transfer of U^FL^ was less effective than that of U^F^; furthermore, ODN2(U^FL^) featuring C-FBs also displayed an effective G-effect and could, therefore, act as an A-selective SNP probe.^19^

## Conclusions

A previously reported quencher-free MB system featuring a 2′-deoxyuridine residue (U^FL^) labeled with a fluorene moiety (separated by an ethynyl linker) and C-FBs could discriminate a perfectly matched DNA target through an enhancement in fluorescence intensity. This discrimination originated from (i) effective ICT between the fluorene and uracil moieties after photoexcitation and (ii) the quenching effect of guanine residues that were complementary to the cytosine bases positioned as the FBs of the U^FL^ residue. As potential alternatives to the ethynyl linker, in this present study we prepared the linker-free U^F^ and the U^DF^ featuring a diacetylenic linker, as well as their corresponding DNA probes. The emission of the isolated probe containing the U^F^ residue was quenched through prominent ICT, but a large increase in fluorescence occurred when it met its matched target. Nevertheless, the fluorene moiety (in the absence of a linker) affected the three-dimensional structure of the mismatched duplexes, such that an effective G-effect was rare, resulting in low discrimination of mismatched targets. On the other hand, the presence of a diethynyl linker twisted the dihedral angle between the fluorene moiety and the uracil base, inhibiting electron transfer between the two moieties, such that its ODNs did not function as probes at all. Overall, the ethynyl linker was most suitable for positioning between the fluorene and uracil moieties. Because the π-conjugated bridge in U^FL^ was quite planar, an electron could be transferred smoothly from the fluorene donor to the uracil acceptor. On the other hand, none of the probes featuring T-FBs exhibited selective fluorescence increases for their matched targets, due to the lack of a G-effect. Thus, the nature of the linker had a great influence on the dihedral angle and fluorescence properties of the nucleosides, as well as their propensity for electron transfer. Therefore, the selection of a suitable linker is one of the most important factors when designing a probe.

## Experimental

### General

All reactions were performed in dry glassware under Ar atmospheres. Analytical thin layer chromatography (TLC) was performed using Merck 60 F_254_ silica gel plates; column chromatography was performed using Merck 60 silica gel (230–400 mesh). Melting points were determined using an Electrothermal IA 9000 series melting point apparatus and are uncorrected. Optical rotations [*α*]_D_ were measured on a Rudolph Research Analytical AUTOPOL I polarimeter. Infrared (IR) spectra were recorded using a JASCO FT/IR-4100 spectrometer. ^1^H, ^13^C and ^31^P NMR spectra were recorded using a Bruker NMR spectrometer (AVANCE III 500 MHz). High-resolution fast atom bombardment (FAB) mass spectra were recorded using a JEOL JMS-700 mass spectrometer, at the Daegu Center of KBSI, Korea. All commercially available chemicals were used without further purification; solvents were carefully dried and distilled prior to use. The synthesis of U^FL^ has been reported previously.^4*f*^

### Synthetic procedures

#### 2′-Deoxy-5-(9*H*-fluoren-2-yl)uridine (U^F^)

2′-Deoxy-5-iodouridine (1; 270 mg, 0.762 mmol), fluorene-2-boronic acid pinacol ester (2; 668 mg, 2.29 mmol), Cs_2_CO_3_ (993 mg, 3.05 mmol), Pd(OAc)_2_ (17.1 mg, 0.0726 mmol), DPPF (84.4 mg, 0.152 mmol) and CuBr (190 mg, 0.762 mmol) were dissolved in distilled DMF (7.6 mL). Argon was bubbled through the solution and then a pump/purge process was performed with the injection of Ar gas. The mixture was stirred at 55 °C for 5 h. After evaporation of the solvent *in vacuo*, the residue was purified through column chromatography (SiO_2_; hexane/EtOAc, 1 : 1) to yield U^F^ (159 mg, 53%): mp > 150 °C dec; [*α*]^13^_D_ = −10.0° (*c* = 1.00 in MeOH); IR (film): *ν* 3364, 3039, 2933, 1659, 1607, 1591, 1461, 1422, 1351, 1275, 1231, 1089, 1017, 833, 734 cm^−1^; ^1^H NMR (500 MHz, CD_3_OD): *δ* 8.23 (s, 1H; H-6), 7.70 (d, *J* = 7.9 Hz, 1H; fluorene-H), 7.65 (s, 1H; fluorene-H), 7.47–7.42 (m, 2H; fluorene-H), 7.25 (t, *J* = 7.4 Hz, 1H; fluorene-H), 7.19 (td, *J* = 7.4, 0.96 Hz, 2H; fluorene-H), 6.27 (t, *J* = 6.6 Hz, 1H; H-1′), 4.37 (q, *J* = 3.6 Hz, 1H; H-3′), 3.80 (q, *J* = 3.1 Hz, 1H; H-4′), 3.81 (s, 2H; fluorene-CH_2_), 3.75–3.64 (m, 2H; H-5′), 2.27–2.23 (m, 2H; H-2′); ^13^C NMR (125 MHz, CD_3_OD): *δ* 167.0, 164.9, 152.0, 145.0, 144.7, 142.7, 142.6, 139.7, 132.9, 128.3, 128.1, 127.9, 126.2, 121.0, 120.7, 116.6, 89.2, 87.0, 72.2, 62.7, 41.8, 37.8; HRMS-FAB (*m*/*z*): [M + H]^+^ calcd for C_22_H_21_N_2_O_5_, 393.1450; found, 393.1447.

#### [4-(9*H*-Fluoren-2-yl)-buta-1,3-diynyl]triisopropylsilane (5)

2-Ethynyl-9*H*-fluorene (4; 447 mg, 2.35 mmol), H_2_NOH·HCl (49.0 mg, 0.705 mmol) and CuCl (35.0 mg, 0.354 mmol) were dissolved in distilled CH_2_Cl_2_ (4.0 mL) and piperidine (580 μL, 5.87 mmol) was added. A solution of (bromoethynyl)triisopropylsilane (3; 1.22 g, 4.67 mmol) in CH_2_Cl_2_ (3.8 mL) was added and then the mixture was stirred at room temperature for 1 h. After evaporation of the solvent *in vacuo*, the residue was purified through column chromatography (SiO_2_; hexane) to yield 5 (470 mg, 54%): mp > 83 °C dec; IR (film): *ν* 3058, 2941, 2890, 2865, 2724, 2361, 2202, 2128, 2097, 2066, 1944, 1800, 1669, 1607, 1455, 1382, 1300, 1227, 1148, 1066, 996, 915, 879, 803, 766, 749, 733, 730, 658, 607, 463 cm^−1^; ^1^H NMR (500 MHz, CDCl_3_): *δ* 7.70 (d, *J* = 7.5 Hz, 1H; fluorene-H), 7.65 (d, *J* = 7.9 Hz, 1H; fluorene-H), 7.61 (s, 1H; fluorene-H), 7.47 (d, *J* = 3.7 Hz, 1H; fluorene-H), 7.46 (d, *J* = 4.2 Hz, 1H; fluorene-H), 7.31 (t, *J* = 6.9 Hz, 1H; fluorene-H), 7.25 (t, *J* = 7.4 Hz, 1H; fluorene-H), 3.81 (s, 2H; fluorene-CH_2_), 1.18 (s, 3H; CH), 1.05 (s, 18H; CH_3_); ^13^C NMR (125 MHz, CDCl_3_): *δ* 143.7, 143.1, 142.9, 140.8, 131.7, 129.2, 127.5, 126.9, 125.1, 120.4, 119.8, 119.4, 89.7, 87.9, 76.5, 74.7, 36.7, 18.6, 11.3; HRMS-FAB (*m*/*z*): [M]^+^ calcd for C_26_H_30_Si, 370.2117; found, 370.2119.

#### 2-Buta-1,3-diynyl-9*H*-fluorene (6)

Tetrabutylammonium fluoride (1.5 mL) was added to a solution of 5 (382 mg, 1.03 mmol) in distilled THF (3.4 mL) and then the mixture was stirred at 0 °C for 30 min. After evaporation of the solvent *in vacuo*, the residue was purified through column chromatography (SiO_2_; hexane) to yield 6 (110 mg, 50%). CAUTION: this compound was unstable and turned into a black insoluble material upon standing: mp > 98 °C dec; IR (film): *ν* 3343, 3251, 3056, 2918, 2769, 2206, 2055, 1896, 1666, 1610, 1465, 1452, 1417, 1392, 1299, 1235, 1198, 1146, 1098, 999, 956, 943, 877, 832, 768, 643, 620, 557 cm^−1^; ^1^H NMR (500 MHz, CDCl_3_): *δ* 7.80 (d, *J* = 7.5 Hz, 1H; fluorene-H), 7.75 (d, *J* = 7.9 Hz, 1H; fluorene-H), 7.70 (s, 1H; fluorene-H), 7.56 (td, *J* = 6.3, 0.55 Hz, 2H; fluorene-H), 7.40 (t, *J* = 6.9 Hz, 1H; fluorene-H), 7.25 (td, *J* = 7.4, 1.2 Hz, 1H; fluorene-H), 3.90 (s, 2H; fluorene-CH_2_), 2.52 (s, 1H; CH); ^13^C NMR (125 MHz, CDCl_3_): *δ* 143.7, 143.2, 140.7, 131.8, 129.3, 127.6, 127.4, 125.2, 120.5, 119.9, 118.8, 76.3, 73.5, 71.3, 68.4, 36.7; HRMS-FAB (*m*/*z*): [M]^+^ calcd for C_17_H_10_, 214.0782; found, 214.0785.

#### 2′-Deoxy-5-[(9*H*-fluoren-2-yl)buta-1,3-diyn-1-yl]uridine (U^DF^)

A solution of 7 (92.0 mg, 0.429 mmol), 2′-deoxy-5-iodouridine (1; 76.0 mg, 0.215 mmol), Pd(PPh_3_)_2_Cl_2_ (15.0 mg, 0.0214 mmol), CuI (4.00 mg, 0.210 mmol) in distilled DMF (1.6 mL) and trimethylamine (0.5 mL) was subjected to 10 cycles of a pump/purge process with the injection of Ar gas. The mixture was then stirred at 55 °C for 5 h. After evaporation of the solvent *in vacuo*, the residue was purified through column chromatography (SiO_2_; CH_2_Cl_2_/MeOH, 30 : 1) to yield U^DF^ (87 mg, 92%): mp > 220 °C dec; [*α*]^13^_D_ = −37.7° (*c* = 1.00 in MeOH); IR (film): *ν* 3404, 3187, 3122, 2951, 2813, 2144, 1721, 1660, 1612, 1465, 1316, 1271, 1099, 1049, 829 cm^−1^; ^1^H NMR (500 MHz, DMSO-*d*_6_): *δ* 11.77 (s, 1H; NH), 8.50 (s, 1H; H-6), 7.96 (d, *J* = 7.8 Hz, 2H; fluorene-H), 7.79 (s, 1H; fluorene-H), 7.63 (dd, *J* = 7.2, 2.5 Hz, 2H; fluorene-H), 7.42 (t, *J* = 7.3 Hz, 1H; fluorene-H), 7.37 (td, *J* = 7.4 Hz, 1.1 Hz, 1H; fluorene-H), 6.10 (t, *J* = 6.4 Hz, 1H; H-1′), 5.27 (d, *J* = 4.3 Hz, 1H; OH-3′), 5.19 (t, *J* = 4.9 Hz, 1H; OH-5′), 4.29–4.25 (m, 1H; H-3′), 3.96 (s, 2H; fluorene-CH_2_), 3.82 (q, *J* = 3.3 Hz, 1H; H-4′), 3.68–3.57 (m, 2H; H-5′), 2.18–2.16 (m, 2H; H-2′); ^13^C NMR (125 MHz, DMSO-*d*_6_): *δ* 161.6, 149.2, 146.2, 143.7, 143.4, 142.7, 140.1, 131.4, 128.9, 127.7, 127.0, 125.3, 120.8, 120.5, 118.3, 96.7, 87.7, 85.2, 82.6, 76.6, 75.7, 78.8, 69.8, 60.7, 40.3, 36.3; HRMS-FAB (*m*/*z*): [M]^+^ calcd for C_26_H_20_N_2_O_5_, 440.1372; found, 440.1373.

#### 5′-*O*-(4,4′-Dimethoxytrityl)-2′-deoxy-5-(9*H*-fluoren-2-yl)uridine (7)

4,4′-Dimethoxytrityl chloride (220 mg, 0.649 mmol) was added to a solution of U^F^ (200 mg, 0.510 mmol) in anhydrous pyridine (200 μL) and then the mixture was stirred under Ar for 10 h at room temperature. The solvent was evaporated and the residue purified chromatographically (SiO_2_, hexane/EtOAc, 1 : 1) to yield 7 (290 mg, 82%): mp > 137 °C dec; [*α*]^13^_D_ = −6.7° (*c* = 1.40 in CHCl_3_); IR (film): *ν* 3476, 3056, 2926, 1691, 1607, 1508, 1461, 1421, 1274, 1249, 1091, 1032, 862, 736 cm^−1^; ^1^H NMR (500 MHz, CDCl_3_): *δ* 8.45 (s, 1H; NH), 7.87 (s, 1H; H-6), 7.71 (d, *J* = 7.5 Hz, 1H; fluorene-H), 7.52 (d, *J* = 7.9 Hz, 1H; fluorene-H), 7.45–7.30 (m, 7H; fluorene-H + DMTr-H), 7.22–7.12 (m, 7H; fluorene-H + DMTr-H), 6.64 (dt, *J* = 8.7, 1.9 Hz, 4H; DMTr-H), 6.44 (t, *J* = 6.0 Hz, 1H; H-1′), 4.48–4.42 (m, 1H; H-3′), 4.10 (q, *J* = 3.7 Hz, 1H; H-4′), 3.63 (s, 6H; OCH_3_), 3.50–3.27 (m, 4H; fluorene-CH_2_ + H-5′), 2.56–2.34 (m, 2H; H-2′); ^13^C NMR (125 MHz, CDCl_3_): *δ* 162.1, 158.5, 149.9, 144.3, 143.5, 143.3, 141.4, 141.2, 136.5, 135.3, 135.4, 130.3, 130.0, 129.8, 129.7, 127.8, 127.0, 126.7, 126.6, 124.9, 119.9, 119.6, 116.3, 113.1, 86.6, 86.3, 85.3, 72.3, 63.3, 55.0, 41.4, 36.5; HRMS-FAB (*m*/*z*): [M]^+^ calcd for C_43_H_38_N_2_O_7_, 694.2679; found, 694.2683.

#### 5′-*O*-(4,4′-Dimethoxytrityl)-2′-deoxy-5-[(9*H*-fluoren-2-yl)buta-1,3-diyn-1-yl]uridine (8)

Using a procedure similar to that described for 7, this product was obtained in a yield of 79%: mp > 180 °C dec; IR (film): *ν* 2929, 2359, 1697, 1607, 1507, 1456, 1277, 1249, 1092, 1033, 825, 733 cm^−1^; ^1^H NMR (500 MHz, DMSO-*d*_6_): *δ* 11.83 (s, 1H; NH), 8.16 (s, 1H; H-6), 7.97 (dd, *J* = 7.9, 4.5 Hz, 2H; fluorene-H), 7.75 (s, 1H; fluorene-H), 7.62 (d, *J* = 7.3 Hz, 1H; fluorene-H), 7.58 (d, *J* = 7.9 Hz, 1H; fluorene-H), 7.43–7.23 (m, 11H; fluorene-H + DMTr-H), 6.90 (t, *J* = 8.8 Hz, 4H; DMTr-H), 6.11 (t, *J* = 6.5 Hz, 1H; H-1′), 5.34 (d, *J* = 4.5 Hz, 1H; H-3′), 4.33–4.28 (m, 1H; H-4′), 3.97 (s, 2H; fluorene-CH_2_), 3.73 (s, 6H; OCH_3_), 3.29–3.14 (m, 2H; H-5′), 2.35–2.22 (m, 2H; H-2′); ^13^C NMR (125 MHz, DMSO-*d*_6_): *δ* 161.5, 158.1, 149.2, 145.5, 144.7, 143.7, 143.5, 142.7, 140.1, 135.6, 135.3, 131.3, 129.7, 129.6, 128.8, 127.9, 127.7, 127.0, 126.7, 125.3, 120.8, 120.5, 118.5, 113.2, 97.1, 86.0, 85.9, 85.5, 82.6, 76.9, 75.1, 73.8, 70.2, 63.6, 55.0, 36.3; HRMS-FAB (*m*/*z*): [M]^+^ calcd for C_47_H_38_N_2_O_7_, 742.2679; found, 742.2682.

#### 5′-*O*-[Bis(4-methoxyphenyl)phenylmethyl]-2′-deoxy-5-(9*H*-fluoren-2-yl)-3′-[2-cyanoethylbis(1-methylethyl)phosphoramidyl]uridine (9)

2-Cyanoethyl *N*,*N*-diisopropylchlorophosphoramidite (77.0 μL, 0.344 mmol) was added dropwise to a solution of 7 (200 mg, 0.288 mmol) and *N*-methylmorpholine (95.0 μL, 0.862 mmol) in CH_2_Cl_2_ (7.2 mL) and then the mixture was stirred at room temperature for 30 min. Evaporation of the solvent *in vacuo* and purification of the residue through short column chromatography (SiO_2_; hexane/EtOAc, 1 : 1) yielded 9 (227 mg, 88%): mp > 80 °C dec; IR (film): *ν* 2960, 2923, 2851, 2358, 1685, 1607, 1509, 1459, 1249, 1177, 1031, 977, 878, 827, 756, 736, 701, 641, 603 cm^−1^; ^1^H NMR (500 MHz, CDCl_3_): *δ* 7.85 and 7.80 (2s, 1H; NH), 7.63 and 7.61 (2s, 1H; H-6), 7.41–7.31 (m, 4H; fluorene-H), 7.30–7.40 (m, 12H; fluorene-H + DMTr-H), 6.60–6.50 (m, 4H; DMTr-H), 6.39–6.32 (m, 1H; H-1′), 4.58–4.52 (m, 1H; H-3′), 4.19–4.23 (m, 1H; H-4′), 3.85–3.74 (m, 1H; OCH_2_), 3.72 (s, 2H; fluorene-CH_2_), 3.70–3.59 (m, 1H; OCH_2_), 3.54 and 3.53 (2s, 6H; OCH_3_), 3.43–3.12 (m, 4H; NCH + H-5′), 2.62–2.49 (m, 2H; CH_2_CN + H-2′), 2.39–2.21 (m, 2H; CH_2_CN + H-2′), 1.20–1.18 (m, 12H, NCHC*H*_3_); ^31^P NMR (202 MHz, CDCl_3_): *δ* 149.1, 148.6.

#### 5′-*O*-[Bis(4-methoxyphenyl)phenylmethyl]-2′-deoxy-5-[(9*H*-fluoren-2-yl)buta-1,3-diyn-1-yl]-3′-[2-cyanoethylbis(1-methylethyl)-phosphoramidyl]uridine (10)

Using a procedure similar to that described for 9, this product was obtained in a yield of 76%: mp > 72 °C dec; ^1^H NMR (500 MHz, DMSO-*d*_6_): *δ* 8.22 and 8.23 (2s, 1H; NH), 7.99 and 7.97 (2s, 1H; H-6), 7.76 (d, *J* = 3.5 Hz, 1H; fluorene-H), 7.62 (d, *J* = 7.4 Hz, 1H; fluorene-H), 7.59–7.56 (m, 2H; fluorene-H), 7.43–7.22 (m, 12H; fluorene-H + DMTr-H), 6.91–6.88 (m, 4H; DMTr-H), 6.11 (m, 1H; H-1′), 4.54–4.48 (m, 1H; H-3′), 4.12–4.01 (m, 1H; H-4′), 3.97 (s, 2H; fluorene-CH_2_), 3.90–3.80 (m, 1H; OCH_2_), 3.75 and 3.74 (2s, 6H; OCH_3_), 3.64–3.54 (m, 3H; OCH_2_ + NCH), 3.27–3.19 (m, 2H; H-5′), 2.80–2.76 (m, 2H; CH_2_CN + H-2′), 2.68–2.64 (m, 2H; CH_2_CN + H-2′) 1.14–0.98 (m, 12H, NCHC*H*_3_); ^31^P NMR (202 MHz, CDCl_3_): *δ* 147.7, 147.3.

### Synthesis of oligonucleotides

ODNs were prepared using the β-cyanoethylphosphoramidite method on controlled pore glass supports (1 μmol) with a POLYGEN Professional 12-Column DNA synthesizer and standard methods.^20^ After automated synthesis, the oligonucleotides were cleaved from the solid support and deprotected through treatment with 30% aqueous NH_4_OH (1.0 mL) for 10 h at 55 °C. The crude products from the automated ODN synthesis were lyophilized and diluted with distilled water (1 mL); they were then purified using high-performance liquid chromatography (HPLC; Grace VyDAC™ C18 column, 250 × 10 mm; pore size: 120 Å). The HPLC mobile phase was held isocratically for 10 min using 5% MeCN/0.1 M triethylammonium acetate (TEAA; pH 7.0) at a flow rate of 2.5 mL min^−1^. The gradient was then increased linearly over 10 min from 5% MeCN/0.1 M TEAA to 50% MeCN/0.1 M TEAA at the same flow rate. The fractions containing the purified ODN were pooled and lyophilized. Aqueous AcOH (80%) was added to the ODN; after standing for 30 min at ambient temperature, the AcOH was evaporated under reduced pressure. The residue was diluted with water (1 mL) and then the solution was purified through HPLC under the same conditions described above. The concentrations of the ODNs were determined through measurement of UV-Vis absorptions. MALDI-TOF mass spectra of the ODNs were recorded using a Kratos Analytical AXIMA LNR MALDI TOF mass spectrometer operated in the linear mode with an 8 : 1 mixture of 3-hydroxypicolinic acid (0.35 M) and ammonium citrate (0.1 M) as the matrix; the accelerating voltage was 20 kV.

### Melting temperatures (*T*_m_)

All values of *T*_m_ of the ODNs (1.5 μM) were recorded in 10 mM Tris–HCl buffer (pH 7.2) containing 100 mM NaCl and 20 mM MgCl_2_. Absorbance–temperature profiles were measured at 260 nm using a Cary 100 Conc UV-Vis spectrophotometer equipped with a temperature controller (cell path length: 1 cm). The absorbance of the samples was monitored at 260 nm upon varying the temperature from 5 to 90 °C at a heating rate of 1 °C min^−1^. Melting temperatures were determined using a derivative method and Cary Win UV thermal application software. Each measurement was run in triplicate.

### UV and fluorescence spectroscopy

ODN solutions were prepared as described above for the measurement of the melting temperatures. Absorption spectra were recorded using a Cary 100 Conc UV-Vis spectrophotometer (cell path length: 1 cm). Fluorescence spectra were recorded using a Cary Eclipse fluorescence spectrophotometer (cell path length: 1 cm; excitation at absorption maximum).

### Optimized structure calculations

To understand absorption and emission phenomena of the nucleosides, the theoretical calculations were carried out by using the software package Gaussian 09 D.^21^ The calculation using B3LYP level with 6-31G basis set provided the optimized geometries at ground states (S_0_) and excited states (S_1_) and the images of the optimized structures were described in ESI.†

## Conflicts of interest

There are no conflicts to declare.

## Supplementary Material

RA-010-D0RA01651A-s001
