# Supplementary material for: The linkers in fluorene-labeled 2′-deoxyuridines affect fluorescence discriminating phenomena upon duplex formation
Source: RSC Adv. 2020 May 19;10(32):18853–9. doi: 10.1039/d0ra01651a (PMC9053879; doi:10.1039/d0ra01651a)
Supplement: RA-010-D0RA01651A-s001 [file RA-010-D0RA01651A-s001.pdf]

Electronic Supplementary Material (ESI) for RSC Advances.
This journal is © The Royal Society of Chemistry 2020

Supplementary information for

The linkers in fluorene-labeled 2′-deoxyuridines affect fluorescence discriminating phenomena upon duplex formation

So Young Lee,[a] Seung Woo Hong,[a] Hyeonuk Yeo*[b] and Gil Tae Hwang*[a]

[a] *Department of Chemistry and Green-Nano Materials Research Center, Kyungpook National University, Daegu 41566, Republic of Korea*
[b] *Department of Chemistry Education, Kyungpook National University, Daegu 41566, Republic of Korea*

Contents

Fig. S1. (a) Absorption and (b) emission spectra of $\mathbf{U^{F}}$, $\mathbf{U^{FL}}$ and $\mathbf{U^{DF}}$ in MeOH at 25 °C S2
Fig. S2. Ground-state optimized molecular structures of (a) $\mathbf{U^{F}}$, (b) $\mathbf{U^{FL}}$ and (c) $\mathbf{U^{DF}}$ S3
Fig. S3. Excited-state optimized molecular structures of (a) $\mathbf{U^{F}}$, (b) $\mathbf{U^{FL}}$ and (c) $\mathbf{U^{DF}}$ S3
Table S1. MALDI-TOF mass spectral data of ODNs containing $\mathbf{U^{F}}$ and $\mathbf{U^{DF}}$ S4
Fig. S4. ^{1}H NMR spectrum of $\mathbf{U^{F}}$ in CD_3OD S6
Fig. S5. ^{1}H NMR spectrum of **5** in $CDCl_3$ S7
Fig. S6. ^{1}H NMR spectrum of **6** in $CDCl_3$ S8
Fig. S7. ^{1}H NMR spectrum of $\mathbf{U^{DF}}$ in DMSO-d_6 S9
Fig. S8. ^{1}H NMR spectrum of **7** in $CDCl_3$ S10
Fig. S9. ^{1}H NMR spectrum of **8** in $CDCl_3$ S11
Fig. S10. ^{1}H NMR spectrum of **9** in $CDCl_3$ S12
Fig. S11. ^{1}H NMR spectrum of **10** in DMSO-d_6 S13

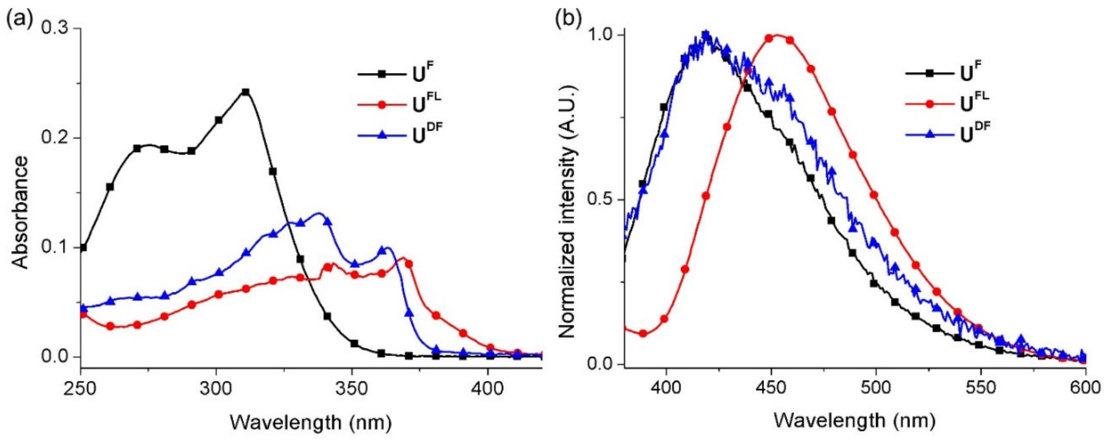

Fig. S1. (a) Absorption and (b) emission spectra of $\mathbf{U^F}$, $\mathbf{U^{FL}}$ and $\mathbf{U^{DF}}$ in MeOH at 25 °C (each at a concentration of 3 μM). Excitation wavelengths: 310 nm for $\mathbf{U^F}$, 369 nm for $\mathbf{U^{FL}}$ and 363 nm for $\mathbf{U^{DF}}$.

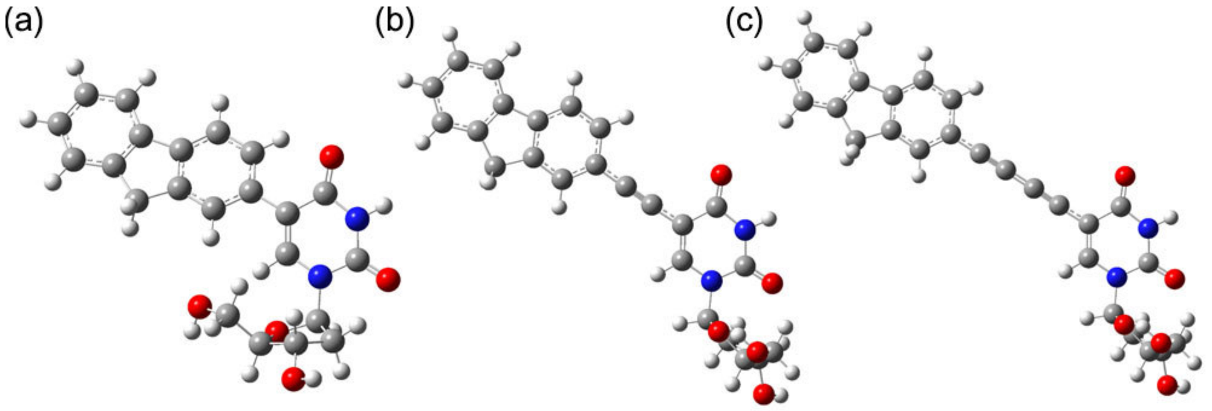

Fig. S2. Ground-state optimized geometries of (a) **U^{F}**, (b) **U^{FL}** and (c) **U^{DF}**, calculated using density functional theory (DFT) at the B3LYP/6-31 level, in vacuo.

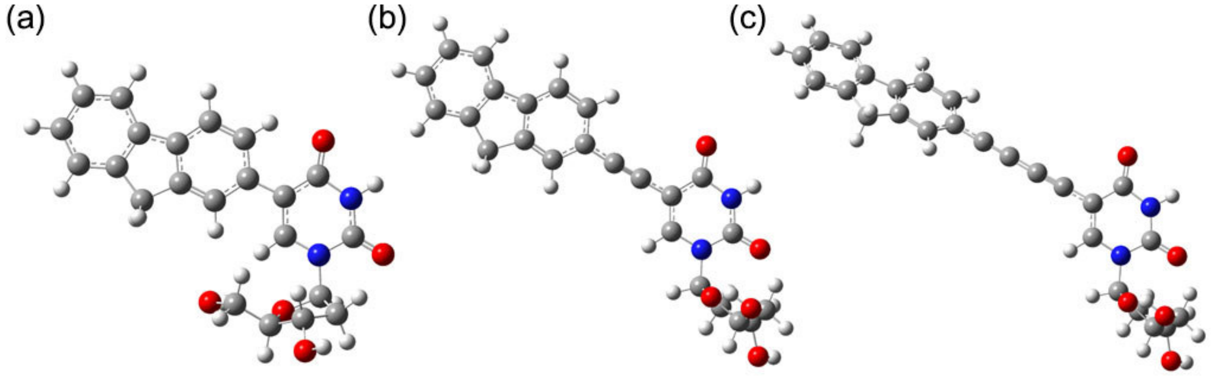

Fig. S3. Excited-state optimized geometries of (a) **U^{F}**, (b) **U^{FL}** and (c) **U^{DF}**, calculated using density functional theory (DFT) at the B3LYP/6-31 level, in vacuo.

Table S1. MALDI-TOF mass spectral data of ODNs containing **U**$^{\mathbf{F}}$ and **U**$^{\mathbf{DF}}$

ODN	Sequence	*m/z* found (calcd) for $[M + H]^+$
ODN1(**U**$^{\mathbf{F}}$)	5′-d(TGG ACT T**U**$^{\mathbf{F}}$T TCA ATG)-3′	4723.5 (4723)
ODN1(**U**$^{\mathbf{DF}}$)	5′-d(TGG ACT T**U**$^{\mathbf{DF}}$T TCA ATG)-3′	4771.5 (4771)
ODN2(**U**$^{\mathbf{F}}$)	5′-d(TGG ACT C**U**$^{\mathbf{F}}$C TCA ATG)-3′	4693.3 (4693)
ODN2(**U**$^{\mathbf{DF}}$)	5′-d(TGG ACT C**U**$^{\mathbf{DF}}$C TCA ATG)-3′	4740.3 (4741)

ODN1(**U**$^{\mathbf{F}}$)

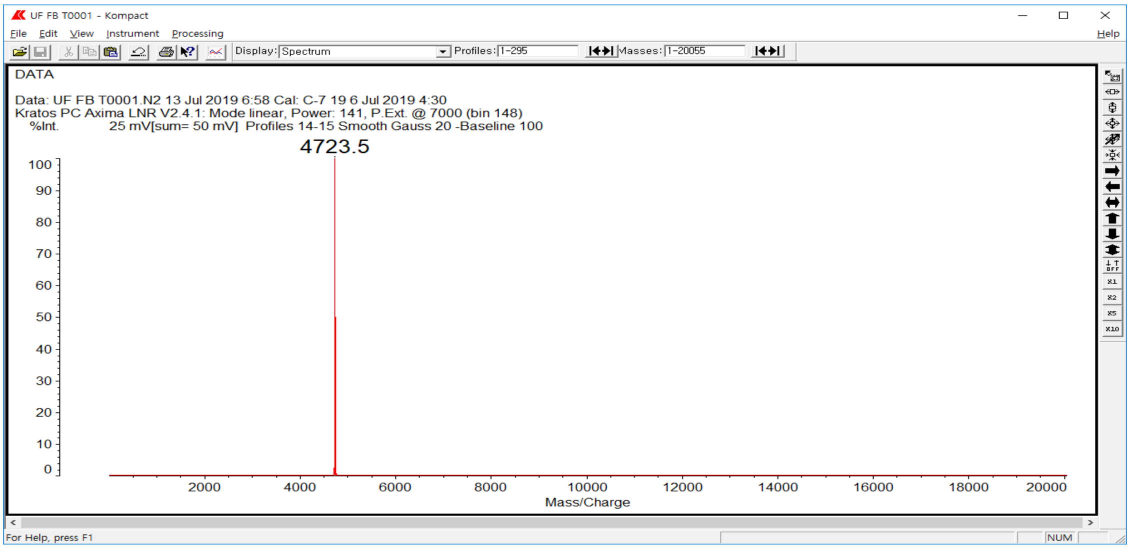

ODN1(**U**$^{\mathbf{DF}}$)

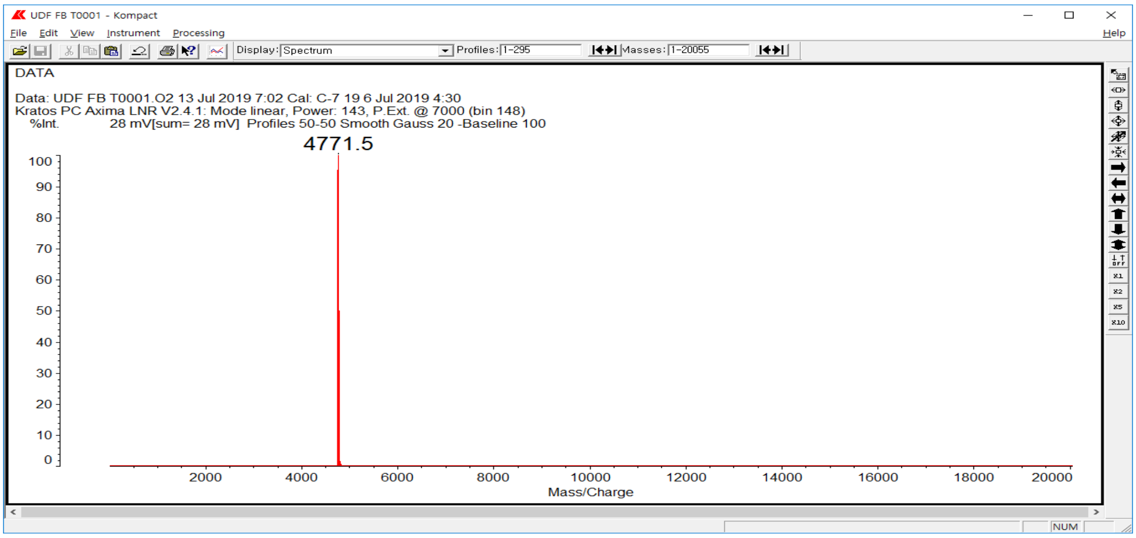

ODN2($\mathbf{U^F}$)

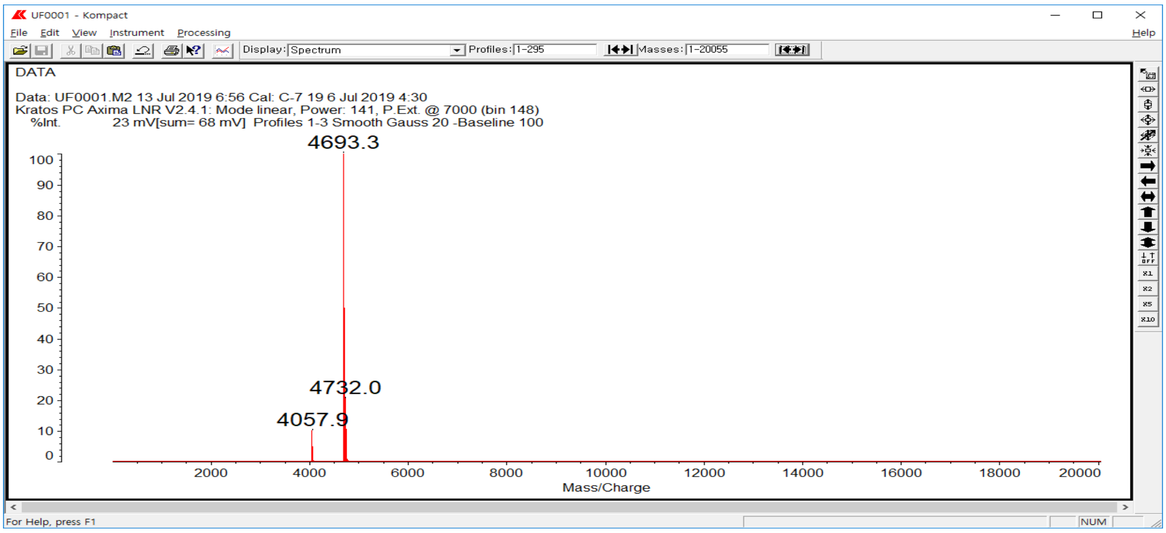

ODN2($\mathbf{U^{DF}}$)

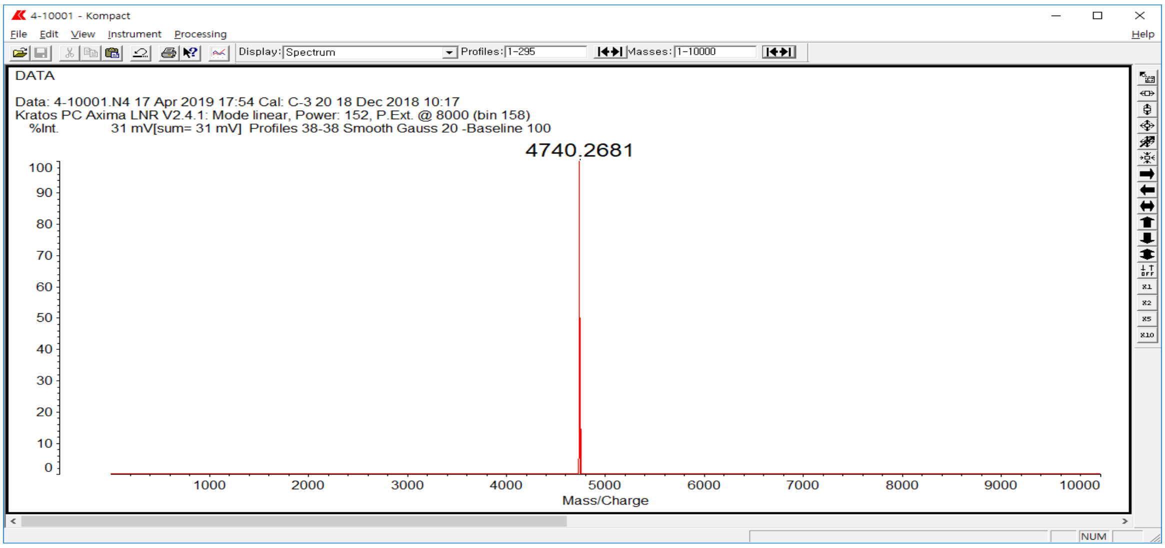

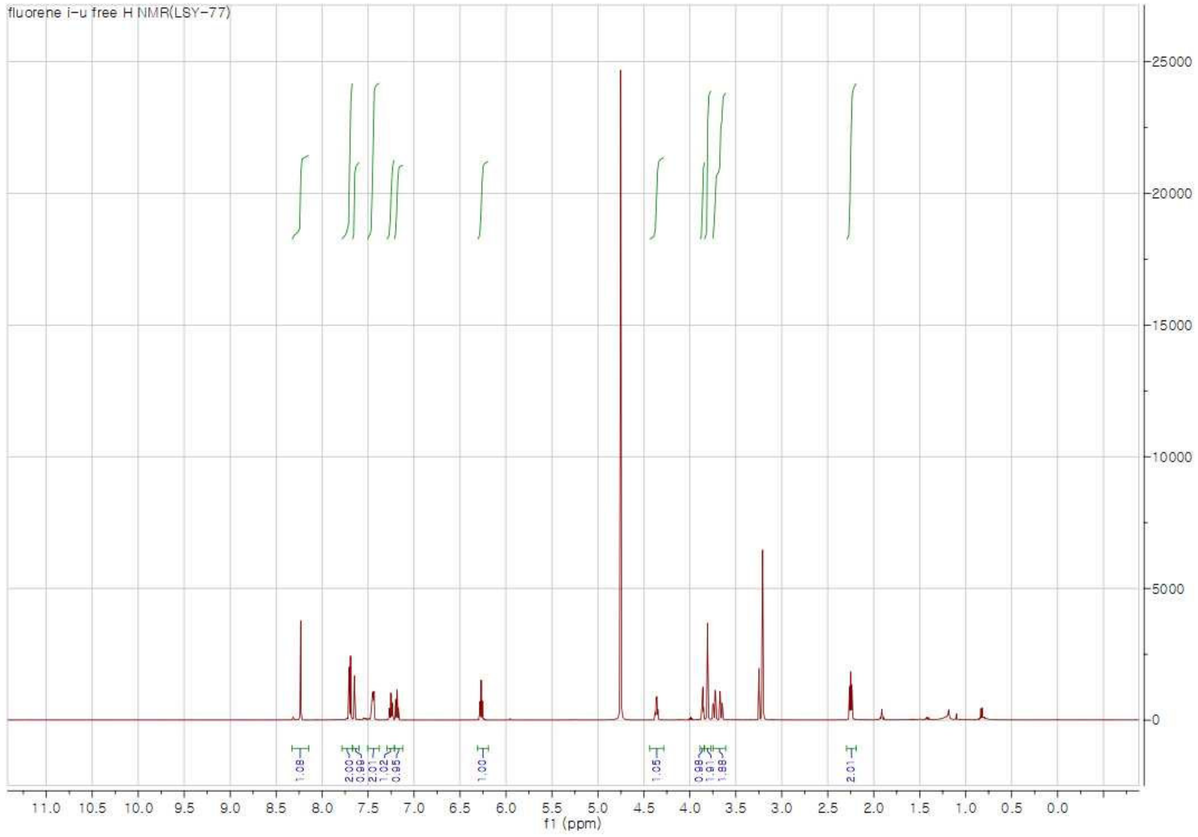

Fig. S4. ^{1}H NMR spectrum of **U^{F}** in CD_3OD.

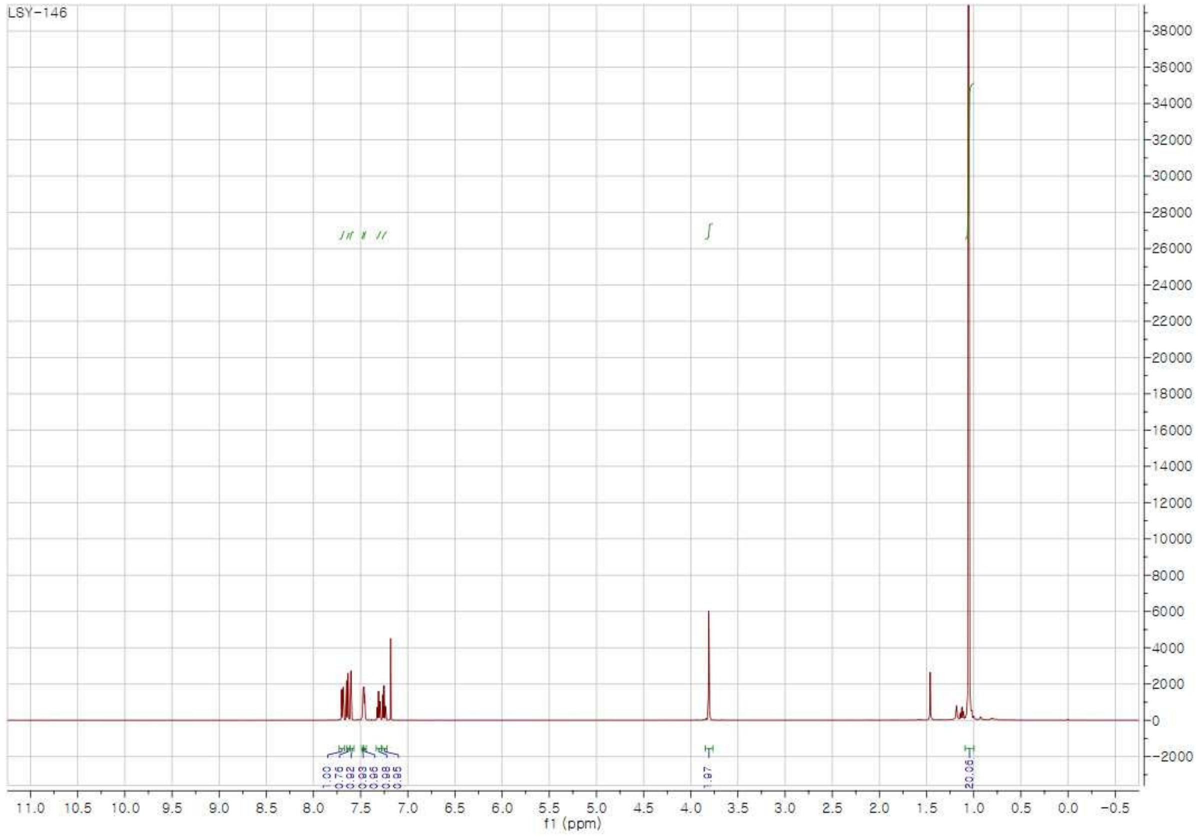

Fig. S5. ^{1}H NMR spectrum of **5** in $CDCl_3$.

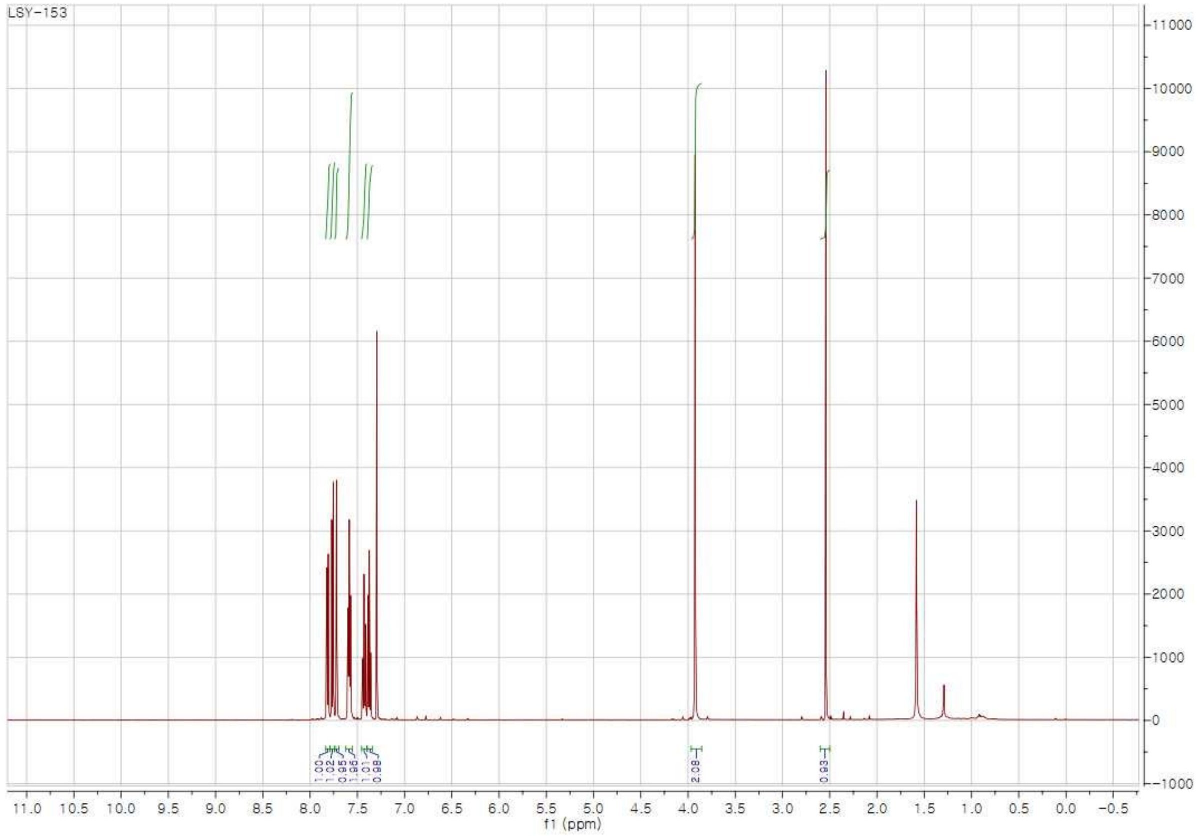

Fig. S6. ^1H NMR spectrum of **6** in $CDCl_3$.

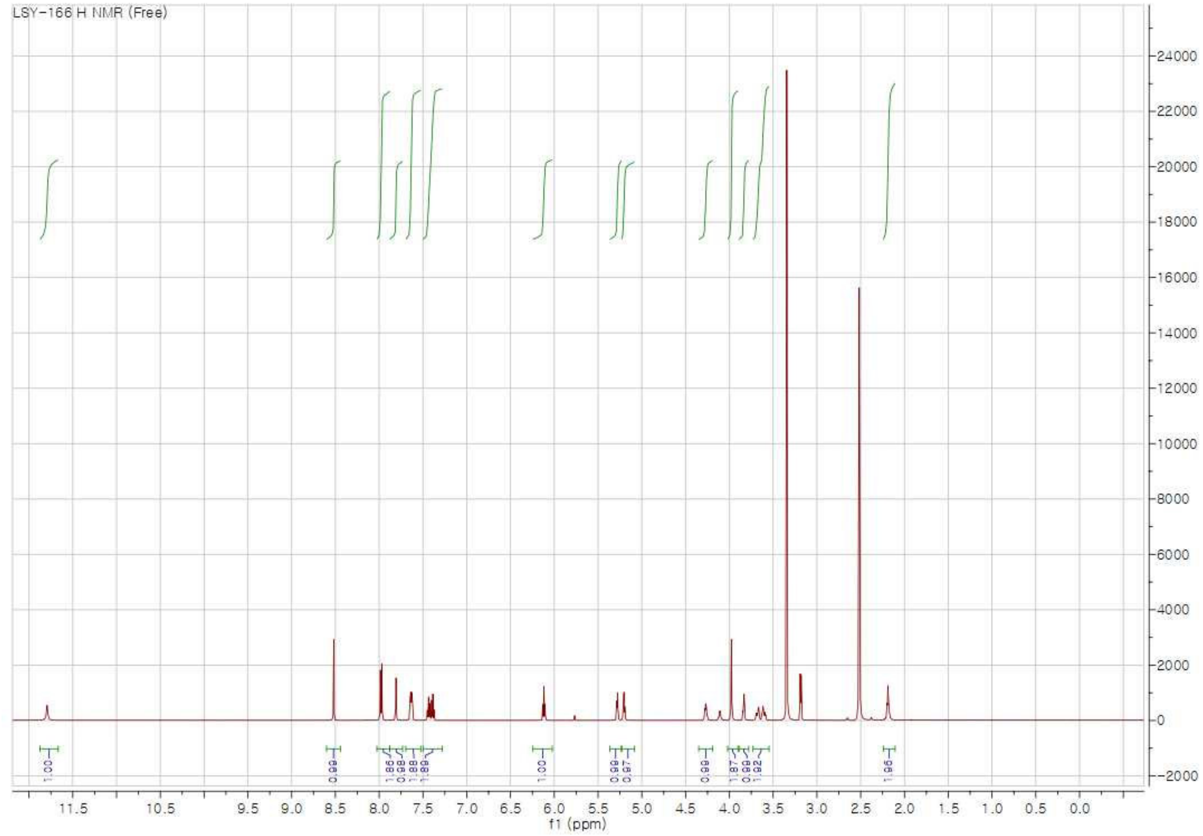

Fig. S7. ^{1}H NMR spectrum of **U^{DF}** in DMSO-d_6.

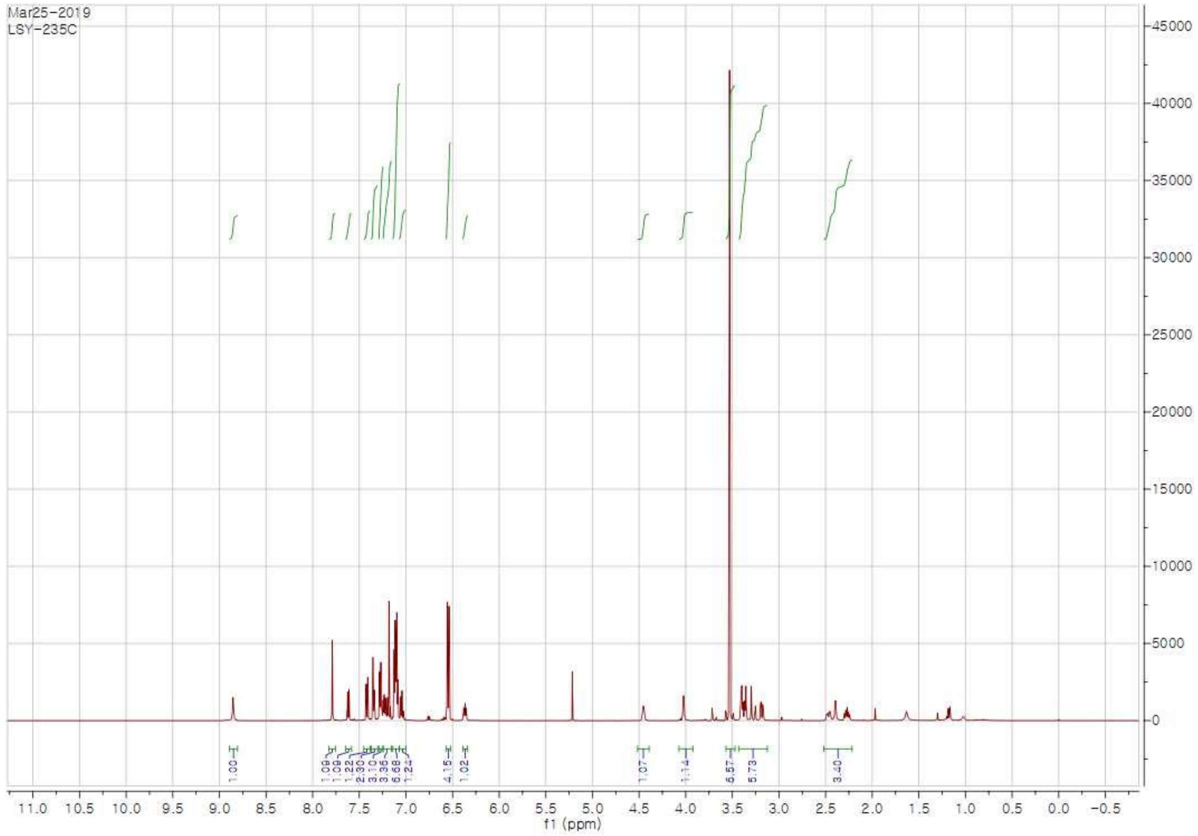

Fig. S8. ^{1}H NMR spectrum of **7** in $CDCl_3$.

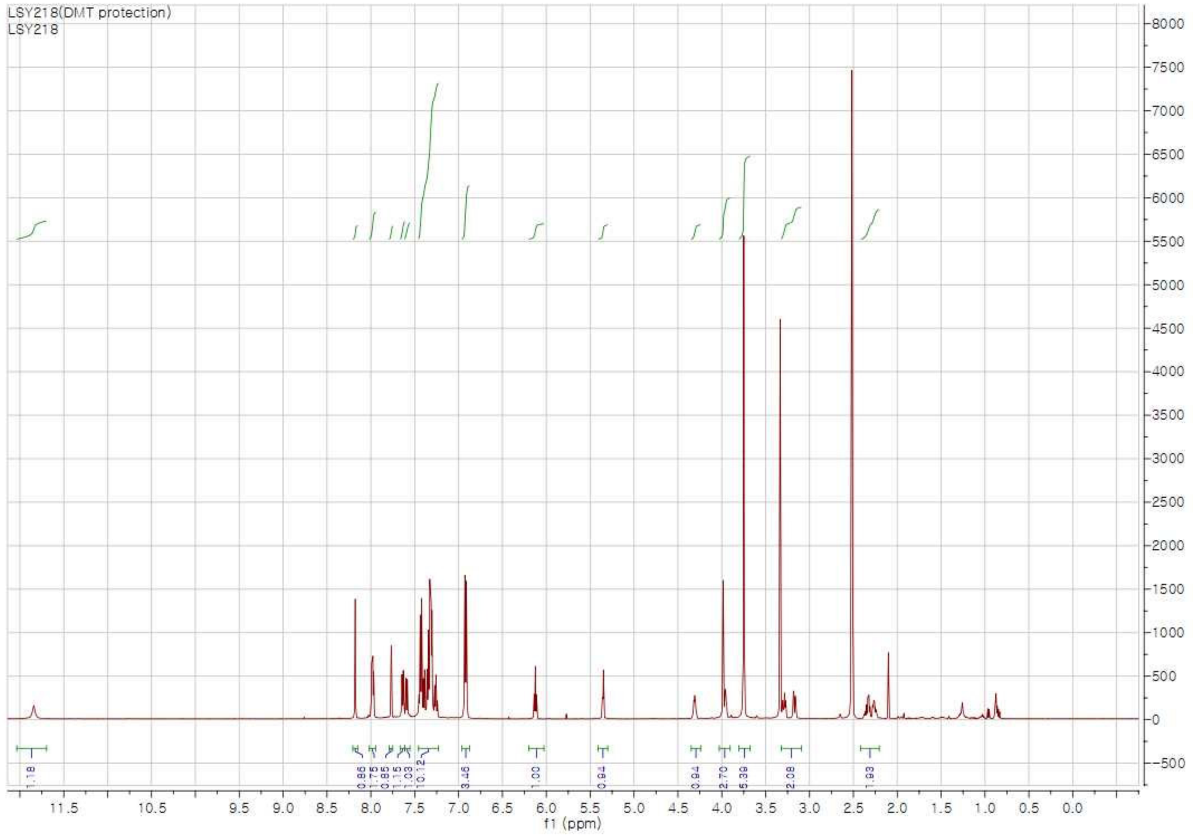

Fig. S9. ^{1}H NMR spectrum of **8** in $CDCl_3$.

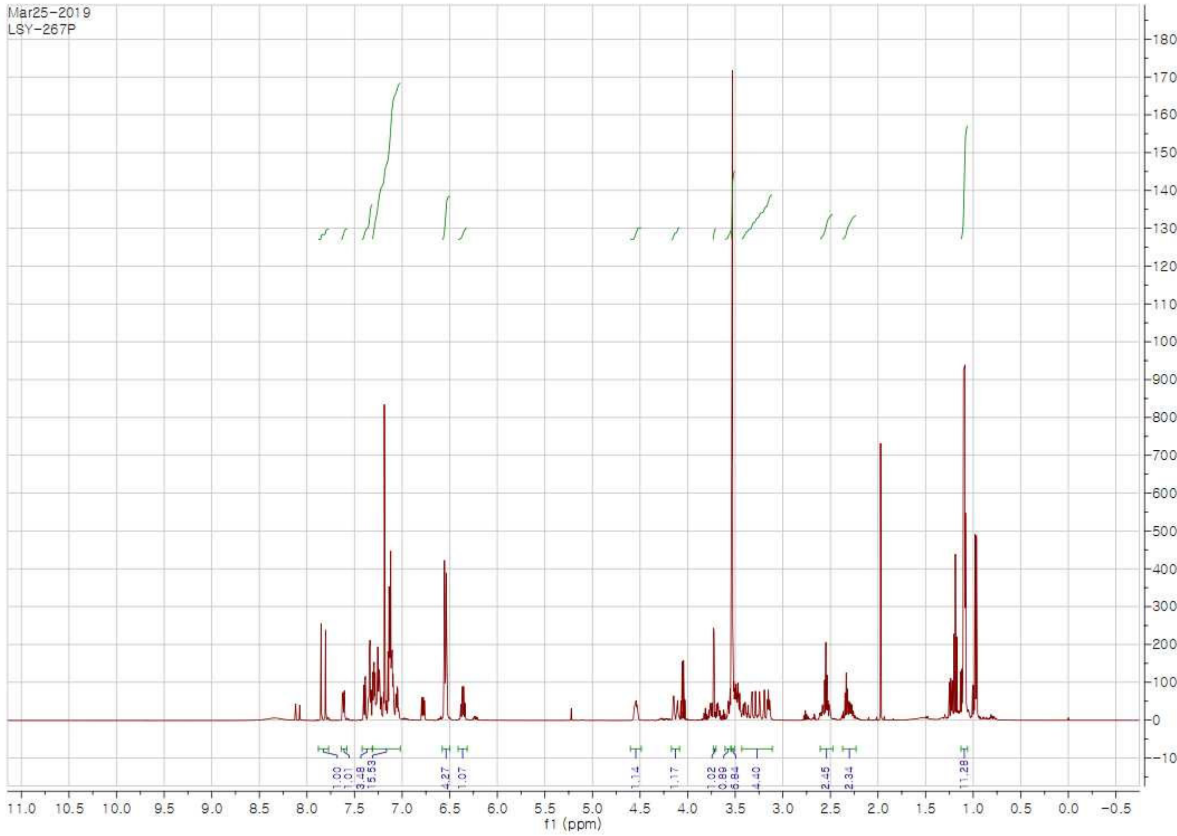

Fig. S10. ^{1}H NMR spectrum of **9** in $CDCl_3$.

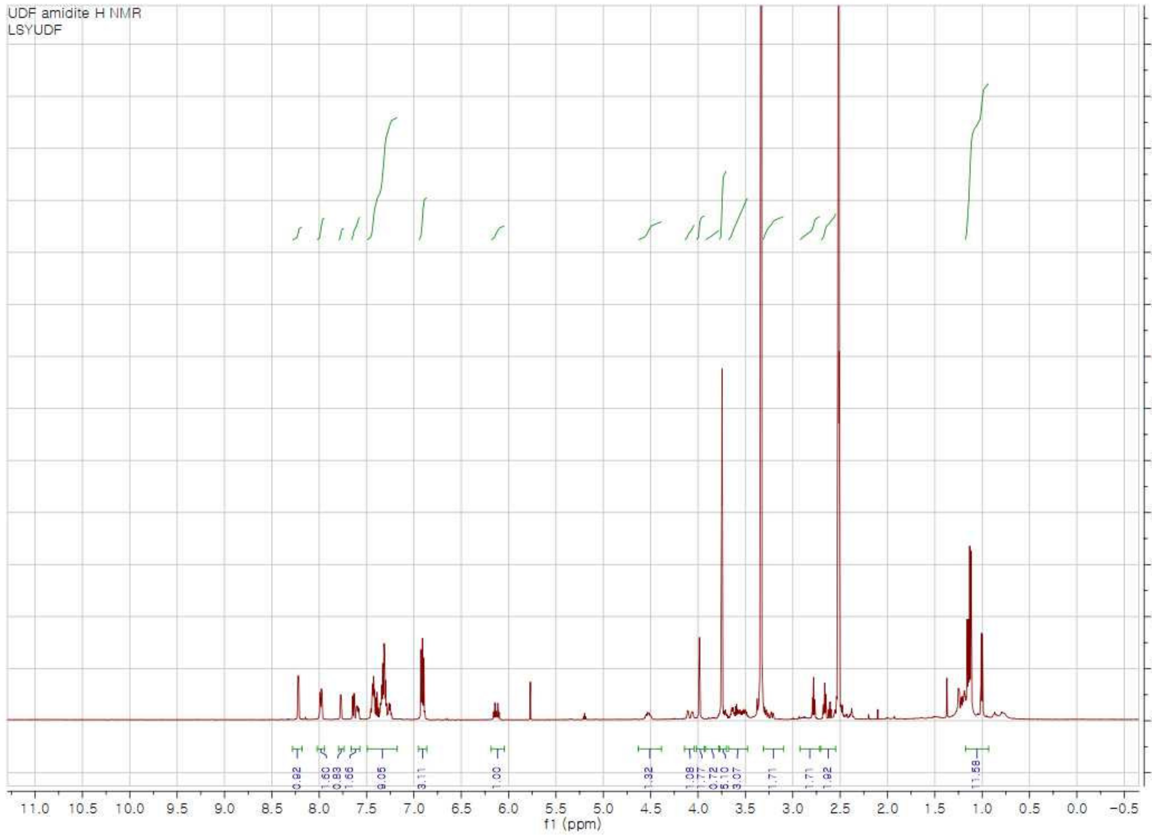

Fig. S11. ^{1}H NMR spectrum of **10** in DMSO-d_6.
